# Paracoccin distribution supports its role in *Paracoccidioides brasiliensis* growth and dimorphic transformation

**DOI:** 10.1371/journal.pone.0184010

**Published:** 2017-08-28

**Authors:** Aline Ferreira Oliveira, Fabricio Freitas Fernandes, Vânia Sammartino Mariano, Fausto Almeida, Luciana Pereira Ruas, Leandro Licursi Oliveira, Constance Oliver, Maria Celia Jamur, Maria Cristina Roque-Barreira

**Affiliations:** 1 Departamento de Biologia Celular e Molecular e Bioagentes Patogênicos, Faculdade de Medicina de Ribeirão Preto, Universidade de São Paulo, Ribeirão Preto, São Paulo, Brasil; 2 Departamento de Biologia Geral, Universidade Federal de Viçosa, Viçosa, Minas Gerais, Brasil; Louisiana State University, UNITED STATES

## Abstract

*Paracoccidioides brasiliensis* yeast was reported to express paracoccin, a GlcNAc-binding protein that displays *N*-acetyl-β-d-glucosaminidase (NAGase) activity. Highly specific anti-paracoccin antibodies have been previously used to examine the localization of paracoccin in yeast and inhibit its growth *in vitro*. In the present study, anti-paracoccin antibodies were used to characterize, by scanning confocal microscopy, the distribution of paracoccin in *P*. *brasiliensis* hyphae, transition forms from hyphae to yeast, and mature yeast. In the mycelial phase, paracoccin was detected mainly in the hyphae tips, where it demonstrated a punctate distribution, and was associated with the cell wall. During the first 48 hours after a temperature shift from 26°C to 37°C, paracoccin expression in the differentiating hyphae was mainly detected in the budding regions, i.e. lateral protrusions, and inside the new daughter cells. There was an increased number of chlamydoconidia that expressed a high concentration of paracoccin on their surfaces and/or in their interiors 72–96 hours after the temperature shift. After 120 hours, yeast cells were the predominant form and their cytoplasm stained extensively for paracoccin, whereas Wheat Germ Agglutinin (WGA) staining was predominant on their exterior walls. After 10 days at 37°C, the interior of both mother and daughter yeast cells, as well as the budding regions, stained intensely for paracoccin. The comparison of mRNA-expression in the different fungal forms showed that PCN transcripts, although detected in all evaluated morphological forms, were higher in hypha and yeast-to-hypha transition forms. In conclusion, the pattern of paracoccin distribution in all *P*. *brasiliensis* morphotypes supports prevalent beliefs that it plays important roles in fungal growth and dimorphic transformation.

## Introduction

*Paracoccidioides brasiliensis* is a dimorphic fungus that causes paracoccidioidomycosis (PCM), the most prevalent systemic mycosis in Latin America, and has a broad geographic distribution that runs from Mexico to Argentina [1, 2].

During *P*. *brasiliensis* infection, the transition from mycelium to yeast cells represents an essential part of the overall virulence strategy of the fungus and is required for the establishment of PCM. The transition is stimulated by the rise in temperature that occurs when the inhaled mycelia or conidia contact the host lungs [3, 4].

This dimorphic fungal transition may be induced *in vitro* by shifting the incubation temperature from 26°C to 36°C (mycelia to yeast) or from 36°C to 26°C (yeast to mycelia) [5]. At 26°C, the fungus is a multicellular saprobiotic mycelium, shaped by filamentous structures that grow by apical extension. At 36°C, the fungus is a rounded yeast with a thick wall from which many daughter cells bud. Since the yeast form is essential for the establishment of PCM [6], the mycelia-to-yeast transition is of particular relevance in the fungal biology and pathogenesis [6, 7]. Using transcriptomic data, the plasticity of gene expression during the morphological transition and the resultant fungal persistence and survival has been demonstrated [8].

The conversion from mycelia to yeast occurs in restricted regions of the mycelium [9], suggesting that the distribution of enzymes involved in the process is not homogeneous throughout the hyphae but localized in particular segments represented by lateral swellings where chlamydoconidia appear [10]. These intermediate structures appear a few days after the temperature shift, when hyphae become ghost-like structures [11–14]. Several authors suggest that chlamydoconidia play a prominent role in the mycelia-to-yeast conversion process [15, 16]. Mature, multibudding yeasts are detected around 10 days after the temperature shift [1]. The transformation process includes the separation of daughter cells from the mother cells, which requires partial chitin degradation in the fungal wall. In *Saccharomyces cerevisiae*, chitin degradation and cell separation have been shown to depend on the action of a periplasmic acidic chitinase [17]. Additionally, it was demonstrated that the CHT3 chitinase gene of *Candida albicans* is functionally homologous to the CTS1 gene of *S*. *cerevisiae* [18], where its deletion results in defective yeast segregation.

We have previously shown that the cell wall of *P*. *brasiliensis* yeast contains an *N*-acetyl-glucosamine- and chitin-binding lectin, named paracoccin (PCN), which also has *N*-acetyl-β-d-glucosaminidase (NAGase) activity [19] and stimulates TNF-α production by macrophages [20]. When *P*. *brasiliensis* yeast cells were cultured in the presence of anti-paracoccin antibodies, fungal growth was reduced, yeast morphology was altered, and the chitin-labeling pattern at the cell wall was modified [21]. These observations gave rise to the hypothesis that paracoccin participates in the remodeling and organization of the yeast cell wall.

Since paracoccin binds to chitin and exerts chitinase activity, we hypothesized that paracoccin participates in the cell wall modification that occurs during the fungal transition from mycelia to yeast. To address this hypothesis, we took advantage of well characterized anti-paracoccin antibodies [21, 22] to examine the localization of paracoccin in hyphae, during the transition from mycelia to yeast, and in the mature yeast. Detailed examination of paracoccin localization was facilitated because sections of frozen fungal forms were obtained using a cryostat and analyzed by scanning confocal microscopy.

## Material and methods

### Ethics statement

BALB/c mice of 6–8 weeks of age were maintained in the animal facility of the Molecular and Cellular Biology Department, Ribeirão Preto School of Medicine, University of São Paulo. Animals were housed in individually ventilated cages, in light-tight cabinets, under a 12 h light-dark cycle and maintained at 20–22°C, under optimized hygienic conditions, and with *ad libitum* access to chow and water. Animal procedures were conducted in accordance with the ethical principles of animal research adopted by the Brazilian Society of Laboratory Animal and approved by the Ethical Committee for Ethics in Animal Research (CETEA) of the Ribeirão Preto School of Medicine, University of São Paulo, under protocol number 145/2005.

### *P*. *brasiliensis* isolate and growth conditions

*P*. *brasiliensis* isolate Pb18 was used in this study. Yeast cultures were grown in YPD medium (1% yeast extract (w/v), 2% peptone (w/v), 2% glucose (w/v), pH 6.5), at 37°C on an orbital shaker and subcultured every 7 days. The fungal mycelium phase (M) was obtained according to the method of [1]. Briefly, yeast cells (Y) were inoculated in 200 mL of the same medium and incubated at 26°C for approximately 10 days with shaking, when the conversion had already occurred. For analysis of mycelium-to-yeast phase transition, cells were transferred to Erlenmeyer flasks containing 200 mL of fresh medium, grown for 10 days at 26°C, and shifted to 37°C for the temperature-induced phase transition. During the cultures, growth curves were performed for each experimental condition, to check cell viability and avoid overgrowth (using the samples at mid exponential phase). Cultures samples were collected at 12, 24, 48, 72, 96, 120 hours, and 10 days of growth for protein extractions and confocal microscopy. For purification of paracoccin, yeast cells were transferred from the solid medium to a liquid medium YPD, and cultured at 37°C under shaking for 14 days. The culture medium was refreshed every 48 hours by harvesting the fungal cells through centrifugation and adding fresh medium, to maintain cell viability.

### Anti-paracoccin antibodies

HIII mice, which are high antibody producers [23], were used to produce anti-paracoccin antibodies as described by [21]. Chicken immunization and isolation of the produced anti-paracoccin IgY antibodies were carried out as previously described [22]. All the assays were done using mouse IgG anti-paracoccin antibody and chicken IgY anti-paracoccin. All antibodies preparations confirmed the reproducibility of the previous assays and the reliability of the obtained data.

### Differential Interference Contrast (DIC) microscopy

For DIC microscopy, *P*. *brasiliensis* cells were processed as previously described [24].

### Confocal microscopy

Samples of mycelial and yeast phase cells at 7 days of culture were collected, as well as mycelial-to-yeast phase transition cells at the times stated above, and analyzed by confocal microscopy. Briefly, the samples in liquid culture were washed with PBS, and the cell suspensions were centrifuged at 5,600 *g*. The pellets were embedded in OCT130 Tissue-Tek (Electron Microscopy Sciences), and the samples were frozen in acetone cooled with dry ice. After freezing the samples were stored at -20°C. Six-micron sections of the frozen samples were obtained using a cryostat (Microm D6900 GmbH, Heidelberg, Germany), and placed on gelatinized glass slides. Before immunostaining, the slides were rinsed three times with PBS and blocked with 3% BSA in PBS for one hour at RT. The samples were incubated with mouse polyclonal anti-paracoccin IgG (150 μg/mL) or with anti-paracoccin IgY (1:100), as specified in the figures legends, diluted in blocking buffer for 2 h, at RT. The samples were rinsed with PBS and incubated for 1 hour in the dark with secondary antibodies: goat anti-mouse IgG conjugated with Alexa 488 or donkey IgG anti-chicken IgY conjugated with Alexa 488, diluted 1:1000 in blocking buffer. For detection of chitin on the cell wall, the samples were incubated with 10 μg/mL of wheat germ agglutinin conjugated to Texas Red^®^-X (WGA, Life Technologies, Molecular Probes, Grand Island, NY) in PBS for 1 h at RT in the dark. For detection of the fungal nucleus, samples were incubated with 2.5 μg/mL of DAPI (4',6-diamidino-2-phenylindole, Molecular Probes). After ten washes with PBS for 5 minutes each, the slides were rinsed in ultrapure water and coverslips mounted with Fluoromount-G (Electron Microscopy Sciences, Hatfield, PA). The samples were examined with a TCS SP5 scanning confocal microscope (Leica Microsystems, Manheim, Germany). Serum from pre-immune HIII (1/250) mice or pre-immune IgY (obtained from chicken eggs before immunization) and secondary antibodies alone were used as controls (S1 Fig). All controls were negative. All experiments were performed at least 3 times for each condition, and similar results were observed.

### PCN mRNA relative expression in different morphological forms of *P*. *brasiliensis*

The gene expression profile of *P*. *brasiliensis* was analyzed in mycelium, yeast, and transition forms (at day 3 during transition process) from mycelium-to-yeast and yeast-to-mycelium. The total RNA from the *P*. *brasiliensis* morphological forms was obtained using *Trizol* (Thermo Fisher Scientific Inc., Waltham, USA). Briefly, cells were harvested, disrupted by grinding in liquid nitrogen, and mixed with Trizol, according to the manufacturer’s instructions. Total RNA was treated with DNase I (Thermo Fisher Scientific Inc.). cDNA was synthesized using 1 μg of total RNA with oligo-dT_12-18_ primer (Thermo Fisher Scientific Inc.) and SuperScript III reverse transcriptase (Thermo Fisher Scientific Inc.), according to the manufacturer’s instructions. Real-time PCR was performed using *Kit Platinum SYBR Green qPCR SuperMix-UDG with ROX* (Thermo Fisher Scientific Inc.), according to the manufacturer’s instructions. The following specific primers were used for PCN-RT: 5´-CCGCCCCTTTGGTGATGT-3´ (sense) and 5´-TCGAAAAGCTCTCCCACTTC-3´ (antisense). To measure the gene expression levels, we used CFX96 real-time PCR detection system (Bio-Rad, Hercules, USA). Fold changes in mRNA expression were calculated using the 2^-ΔCq^ formula, where ΔCq is the difference in the threshold cycle (*Cq*) between the target gene and the β-actin (Sequence ID: ref |XM_010763641.1|) and α-tubulin references genes [25]. The reaction of the genes β-actin and α-tubulin was done using a couple of primers: 5′-GGATGAGGAGATGGATTATGG-3′ (sense) and 5′-GAAACACTCGACGCACACGAC-3′ (antisense); and 5′- GTGGACCAGGTGATCGATGT-3′ (sense) and 5′-ACCCTGGAGGCAGTCACA-3′ (antisense), respectively.

## Results

### Paracoccin detection in the *P*. *brasiliensis* hyphae

**Fig 1:**
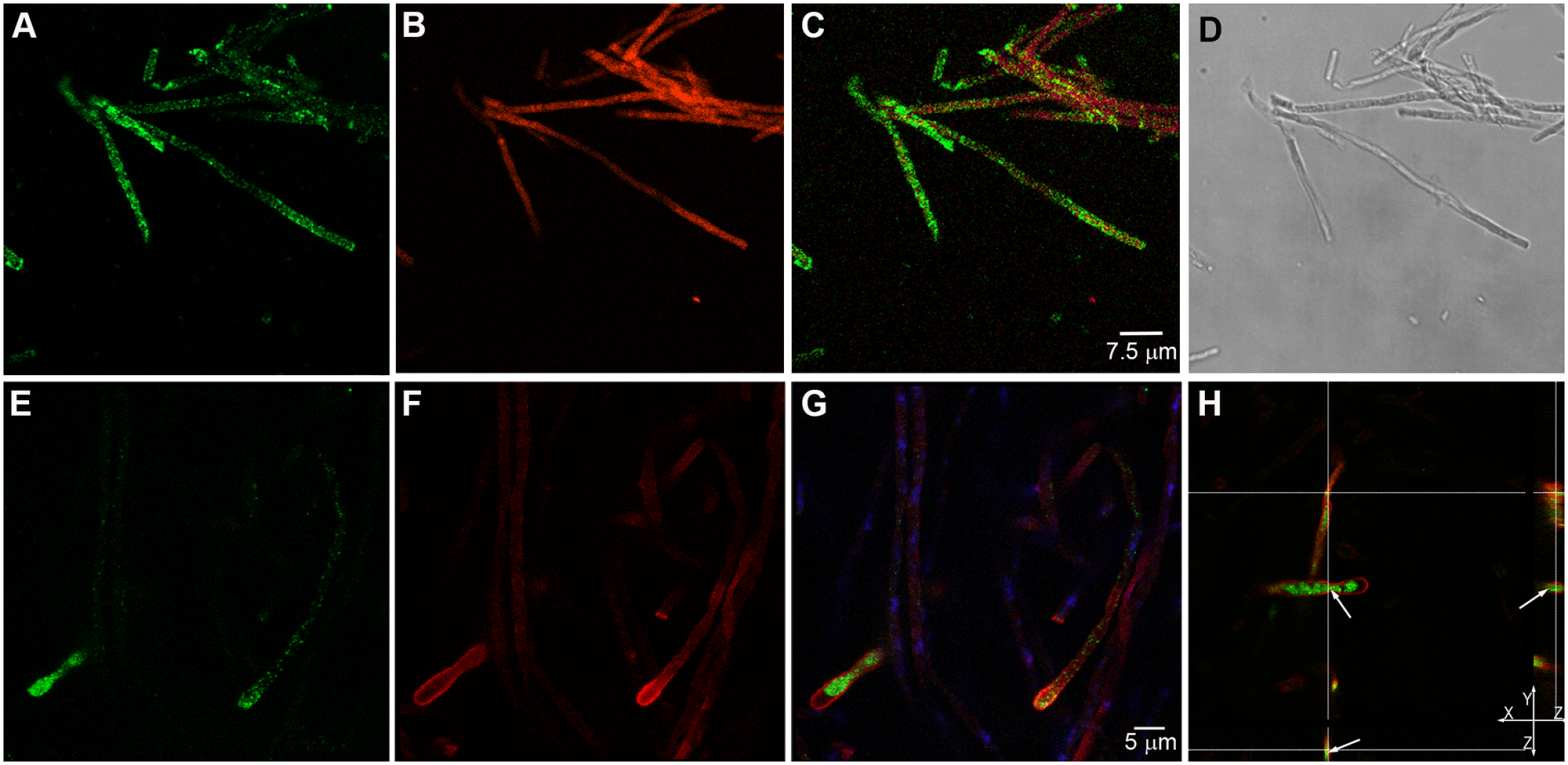
Localization of paracoccin in *Paracoccidioides brasiliensis* hyphae. *P*. *brasiliensis* mycelia were immunostained for paracoccin with chicken IgY anti-paracoccin antibody conjugated to Alexa Fluor 488 (green) (A and E), and stained for chitin with Texas Red^®^-X WGA (red) (B and F), and for DNA with DAPI (blue). Merged images stained for PCN and WGA (C and G), as well as orthogonal (XY, XZ, and YZ) analysis (H) confirmed the localization of paracoccin (arrows). Images A–C are the maximum projection of merged Z series stack and images E–G are a single section from a Z series stack. (D) DIC microscopy of the stained hyphae. (H) Orthogonal analysis of a single section from a Z series stack.

Paracoccin detection in *P*. *brasiliensis* hyphae was carried out by confocal microscopy, using specific anti-paracoccin antibodies (Fig 1). Prior to immunostaining the expected morphological features of the hyphae were confirmed by DIC microscopy. Hyphae immunostained for paracoccin showed a punctuated labeling (Fig 1A and 1E), which was more prominent in the cell tip, where fungal growth is known to occur (Fig 1E). Because paracoccin was previously demonstrated to interact with chitin, we used a β-1,4-GlcNAc-binding lectin (WGA), conjugated to Texas Red^®^-X, to label the chitin in the hyphae. Chitin was distributed in the cell wall (Fig 1B) and, in some hyphae, heavier labeling was detected at the hyphal tips (Fig 1F). In some hyphae, paracoccin and chitin labeling were visualized distinctly, whereas, in others, the punctate paracoccin labeling was closely associated with the chitin labeling, mainly near the hyphal tip, where paracoccin and chitin are more concentrated (Fig 1C and 1G). The orthogonal analysis of a z-slice (Fig 1H) defined the PCN localization inside the hyphae and on their surfaces.

### Paracoccin detection in hyphae-to-yeast transformation

**Fig 2:**
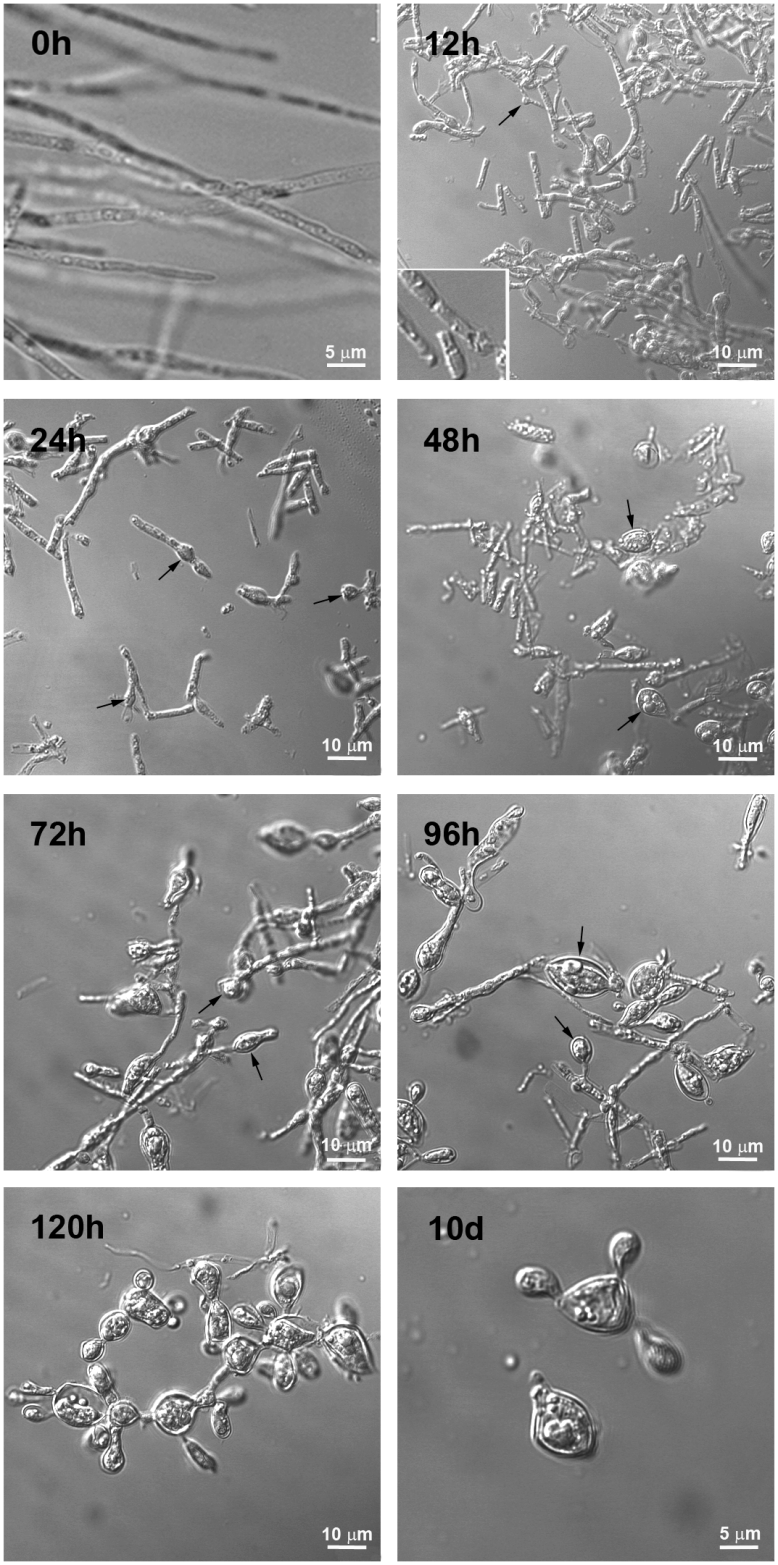
Time-lapsed transformation of *P*. *brasiliensis* hyphae to yeast forms. Mycelia cultured in liquid medium were induced to undergo transformation from hyphal to yeast forms by shifting the temperature from 26°C to 37°C. The images were obtained at 0 h, 12 h, 24 h, 48 h, 72 h, 96 h, 120 h, and 10 days after temperature shift, by DIC microscopy. Budding regions and lateral protrusions are indicated by arrows.

To evaluate the presence of paracoccin during the mycelium-to-yeast transition, the temperature of fungal cultures was raised from 26°C to 37°C and samples were harvested at 12 h intervals from 12 h to 120 h and at 10 days after the temperature shift (Fig 2). Analysis of the morphological changes showed that at 0 h, the hyphae exhibited a multicellular filamentous form that appears as a chain of long and slender cells. The hyphal tips were rounded. Twelve hours after the temperature shift, some hyphae showed small lateral swellings (bud-like protrusions). At that time point, the septa of the mycelial form were more prominent. At 24h after the temperature shift, some hyphae presented tumefactions and had an increased number of tip and lateral swellings, correspondent to chlamydoconidia-like cells or bud-like protrusions. These chlamydoconidia-like cells or bud-like protrusions had enlarged and were more frequently seen at 48 h. At 72 and 96 h chlamydoconidia-like structures were still seen. After 120 h at 37°C, mature, multibudding yeasts were the predominant morphotype. After 10 days in culture, at 37°C, the transition had reached completion and mature budding yeasts were the only morphotype seen. *P*. *brasiliensis* yeasts grew as spherical or oval cells of variable size. The entire structure had the typical steering wheel morphology, reflecting the pattern of multiplication of yeast cells by polar or multipolar budding, in which the buds remain transitorily connected to the mother cell by narrow necks.

**Fig 3:**
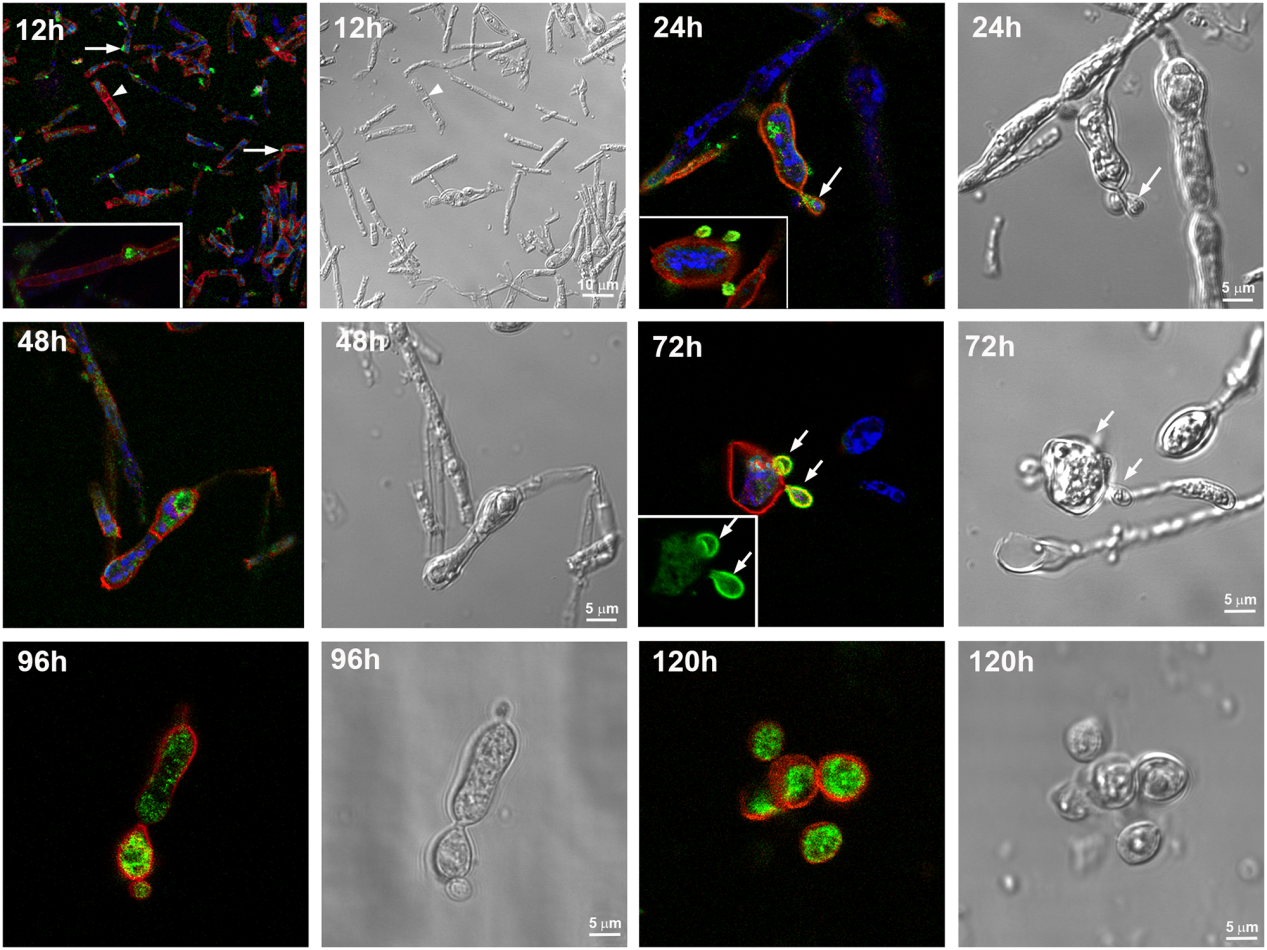
Localization of paracoccin and chitin in *Paracoccidioides brasiliensis* during the transition of mycelium to yeast cells. Mycelia cultured in liquid medium were induced to undergo yeast transformation by shifting the temperature from 26°C to 37°C. Samples were harvested at 12 h, 24 h, 48 h, 72 h, 96 h, and 120 h after the temperature shift and immunostained for paracoccin (PCN), using chicken IgY anti-paracoccin antibody conjugated to Alexa Fluor 488 (green) and stained with WGA (red) for chitin, and with DAPI (blue) for DNA. Budding regions and lateral protrusions are indicated by arrows. A septum is indicated by an arrowhead.

**Fig 4:**
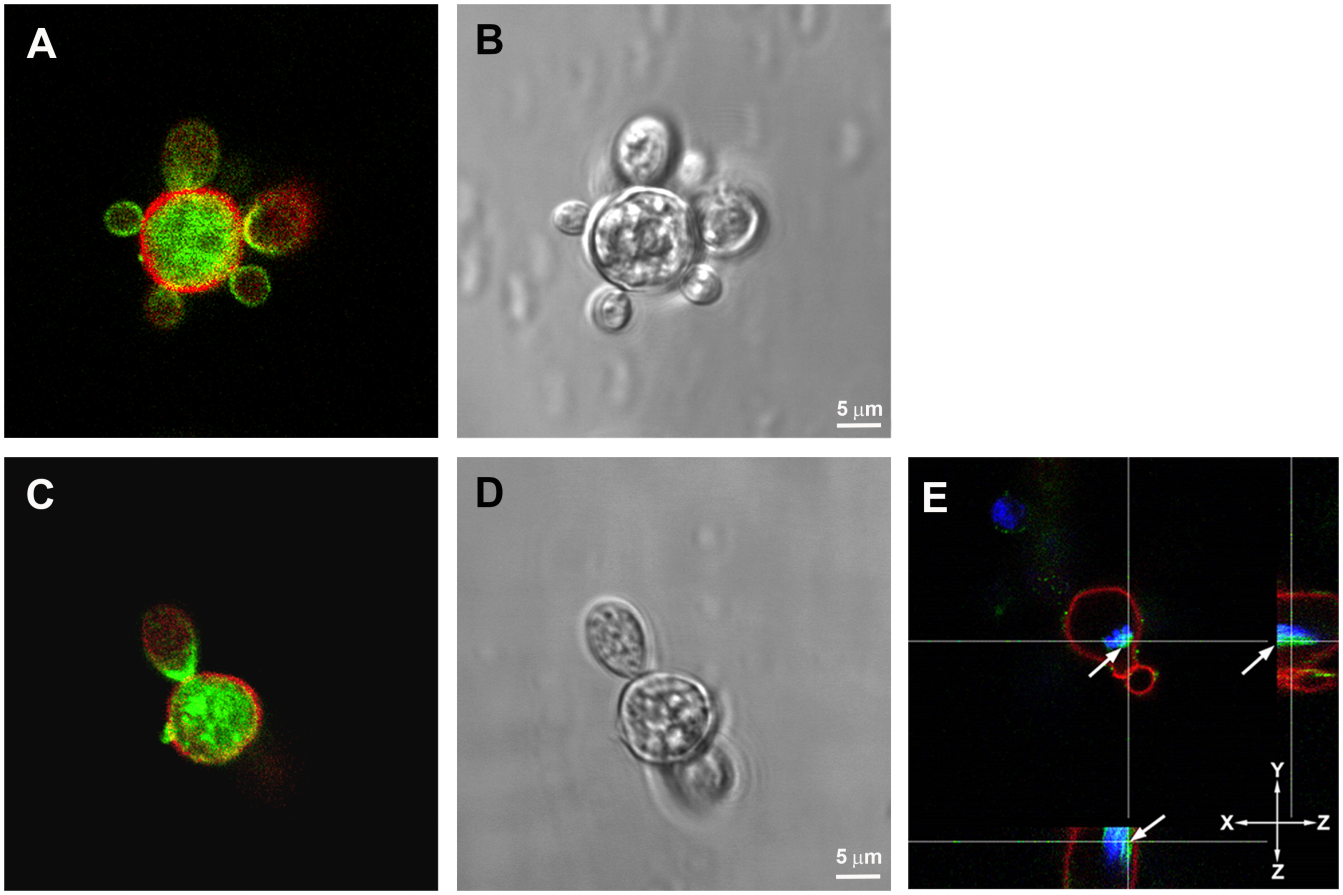
Localization of paracoccin and chitin in yeast cells of *Paracoccidioides brasiliensis*. After 10 days cultured at 37°C, *P*. *brasiliensis* yeasts were stained for detection of paracoccin, using chicken IgY anti-paracoccin antibody conjugated to Alexa Fluor 488 (green), and for chitin with Texas Red^®^-X WGA (red). (E) Orthogonal analysis confirmed the localization of paracoccin and WGA. The orthogonal (XY, XZ and YZ) analysis was performed to confirm the localization of paracoccin and chitin in the yeasts. DNA was stained with DAPI (blue). The images are a single section from a Z series stack.

**Fig 5:**
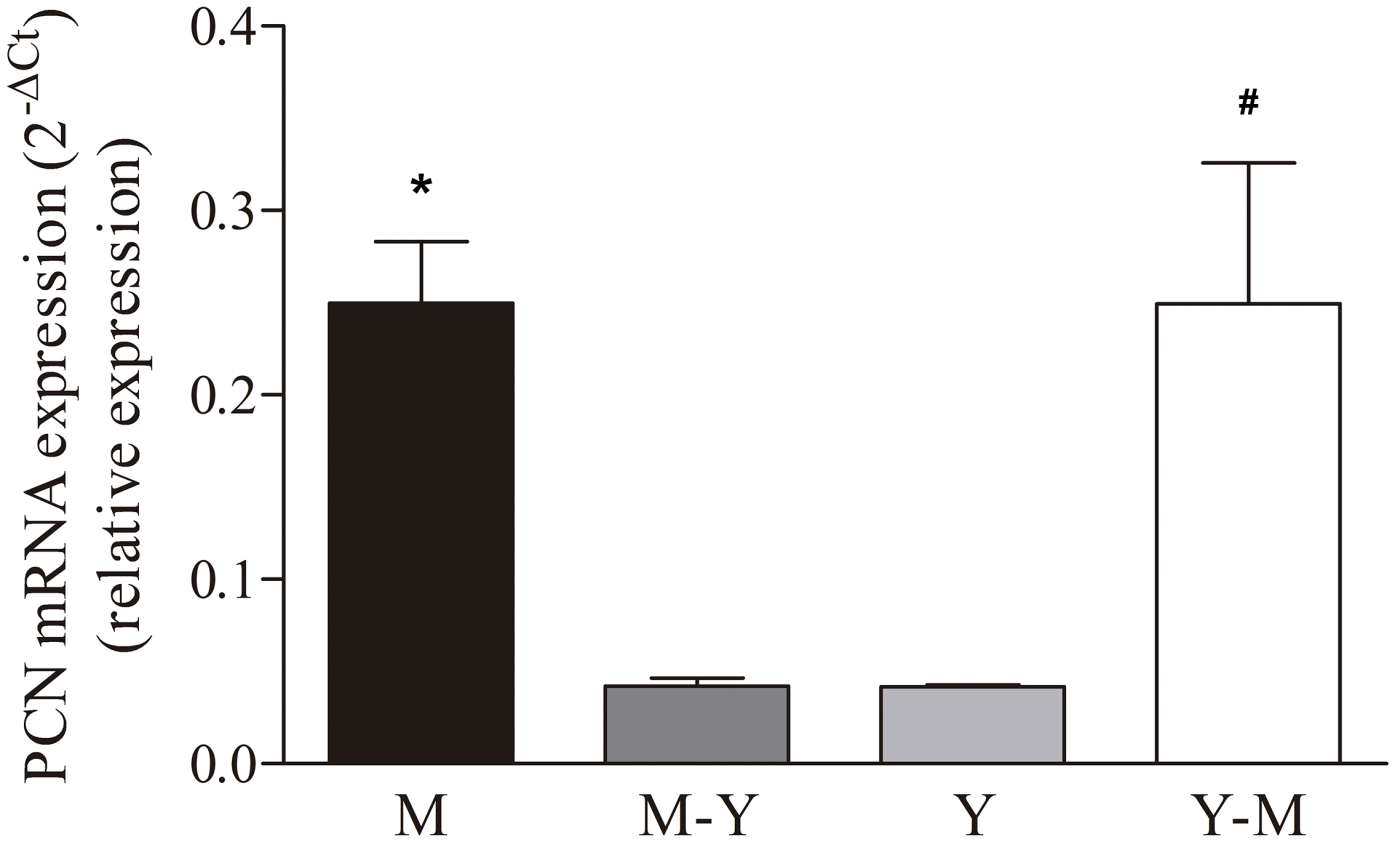
Differential expression of PCN mRNA in different morphological forms of *P*. *brasiliensis*. Evaluation of PCN mRNA differential expression by RT-qPCR using cDNA from mycelia (A), mycelia-to-yeast (B), yeast (C), and yeast-to-mycelia (D). The β-actin and α-tubulin reference genes were used as endogenous controls to normalize the relative PCN mRNA expression. The results are expressed as mean ± SEM; * indicates p < 0.05 between mycelia compared to mycelia-to-yeast or compared to yeast. # indicates p < 0.05 between yeast-to-mycelia compared to mycelia-to-yeast or compared to yeast.

The distribution of paracoccin and chitin in hyphae, during the mycelium-to-yeast transition, and in yeast cells was analyzed by scanning confocal microscopy (Figs 3 to 5). During all the periods analyzed, chitin, detected by using WGA, was localized in the cell wall, including the septa, while paracoccin was distributed in a punctate manner in the cells. At 12 h after the temperature shift, a higher concentration of paracoccin was associated with the lateral protrusions that are related to the fungal growth (Fig 3 and S2 Fig—panel A).

Fungi harvested 24 h after the temperature shift, showed that chlamidoconidia-like cells were labeled with both anti-paracoccin antibody and WGA. Moreover, the budding regions were intensely labeled for paracoccin, predominantly in the new daughter cells. At 48 h after the temperature shift, the staining pattern was like that observed at 24 h, with tumefacted hyphae, and more intense paracoccin staining in the regions related to fungal growth (Fig 3 and S2 Fig—panels D and G).

Fungal forms harvested after 72 h and 96 h at 37°C showed a higher frequency of chlamydoconidia, whose cytoplasm and / or surface was immunolabeled for paracoccin (Fig 3 and S3 Fig—panels A and D). At 72 h, the new daughter cells were intensely stained for paracoccin, which was co-localized with chitin in many cells. On the other hand, at 96 h after the temperature shift, the paracoccin expression was more prominent inside the cells.

In samples harvested from cultures incubated for 120 h at 37°C, there was intense paracoccin labeling in the interior of the yeast forms, while WGA staining was limited to the exterior walls (Fig 3 and S3 Fig—panels F and G).

### Paracoccin detection in yeast cells

Ten days after cultured at 37°C, when the transition was complete, yeast cells had a typical morphology, as detected by Differential Interference Contrast (DIC) (Figs 2 and 4). There was paracoccin labeling in the interior of the mother and daughter cells, and in the budding regions (Fig 4 and S4 Fig). WGA labeling was restricted to the cell walls and, in some yeasts, it was co-localized with paracoccin. Orthogonal analysis (Fig 4E) confirmed that the paracoccin was in the interior of the yeast cell in a juxtanuclear position and that the WGA was restricted to the cell walls.

### Paracoccin expression

Considering the PCN involvement in the morphological transition process of *P*. *brasiliensis*, we evaluated if there were differences in the relative expression of PCN mRNA in yeast, hypha, and transition forms. We compared the normalized mRNA-expression levels detected in the different fungal forms. PCN transcripts were detected in all evaluated morphological forms. Higher expression was verified in hypha and yeast-to-hypha transition forms in comparison to the expression in yeast and hypha-to-yeast transition forms (Fig 5).

## Discussion

We have previously shown that the cell wall of *P*. *brasiliensis* yeast contains an *N*-acetyl-glucosamine- and chitin-binding lectin, named paracoccin (PCN), which also has *N*-acetyl-β-d-glucosaminidase (NAGase) activity [19] and stimulates TNF-α production by macrophages [20]. When *P*. *brasiliensis* yeast cells were cultured in the presence of anti-paracoccin antibodies, fungal growth was reduced, yeast morphology was altered, and the chitin-labeling pattern at the cell wall was modified [21]. These observations gave rise to the hypothesis that paracoccin participates in the remodeling and organization of the yeast cell wall.

Fungal adaptation to the host environment is associated with changes in the cell wall, which include structural alterations in carbohydrate polymers [1]. As the fungus adopts the yeast form, chitin levels increase in the cell wall and is accompanied by a shift in the glucan anomeric structure from a β-1,3-linked polymer to an α-1,3-glucan [26]. In mycelia, only β-linked glucans are present, whereas in yeast, 95% of the glucans are α-linked and 5% β-linked [27]. In the mycelial cell wall, fibers of chitin are interwoven with fibers of β-glucan [9, 28], whereas in the yeast cell wall, chitin and α-glucan are distributed in two distinct layers [28].

We investigated the mycelium-to-yeast transition forms of *P*. *brasiliensis* because, depending on their distribution, molecules expressed during this period may play distinct roles in the fungal transformation process and influence the pathogenesis of paracoccidiodomycosis [8]. For example, the unbranched homopolymer of 1,4-β-linked GlcNAc, i.e., chitin, is abundant in yeasts, especially in the septum that delimits mother and budding cells, indicating that its synthesis and degradation need to be finely regulated by the cell [29, 30]. High levels of chitin are associated with the parasitic phase and cell wall thickness of *Candida albicans* hyphae and *P*. *brasiliensis* yeasts [31, 32]. Our finding of paracoccin localization to regions of growth in the various fungal morphotypes suggested that paracoccin could be involved in the process of *P*. *brasiliensis* transition from mycelial to yeast form. Indeed, we have shown recently that silencing the gene encoding paracoccin results in the development of yeasts that are deficient in cell segregation and are unable to do the transition from yeast to mycelia [33].

We validated the model used in this study by kinetically analyzing the morphological features of *P*. *brasiliensis* following temperature shift. The features we observed were consistent with both original reports [13, 28, 34] and more recent descriptions [35–38] of the fungal morphotypes. As expected, hyphae showed a typical filamentous branching structure with growth tips [39]. The transition forms arose from the hyphae about 24 h after the temperature shift, as indicated by the presence of chlamydoconidia, whose features were also like those in previous reports [13, 15]. We observed that transformation of hyphae to yeast occurred as a continuous process in which chlamydoconidia became progressively more prevalent. Four days after the temperature shift, the yeast form predominated and many multibudding yeast cells were observed, in accordance with previous findings by [35].

All the examined fungal forms displayed paracoccin, as detected by immunofluorescence staining. Inside mycelia, the fine punctate labeling was more concentrated near the tip; this paracoccin labeling was partially coincident with chitin detection, a finding consistent with our previous demonstration that paracoccin interacts with chitin [19, 21, 24]. Localization in the tips suggests that the paracoccin-chitin interaction may be implicated in the hyphal growth, especially taking into consideration that paracoccin has NAGase activity [19]. Hyphal growth is known to be extremely polarized and to require a constant stream of membrane-enclosed secretory vesicles to deliver the molecules necessary to hyphal tip growth. This was demonstrated for *Candida albicans*, whose vesicles contain enzymes implicated in the biosynthesis of the new cell wall [40, 41]. In this case, polarization within the hyphae is strongly dependent on the organizing center, denoted Spitzenkörper, for hyphal growth and morphogenesis [39, 40, 42, 43]. Although the punctate distribution of paracoccin inside the hyphal tips suggests that it could be one of the enzymes contained in the secretory vesicles, we could not detect a structure in *P*. *brasiliensis* hyphae as well defined as the Spitzenkörper in *C*. *albicans*.

It is known that during the transformation of *P*. *brasiliensis* from hyphae to yeast, cracking of the outer electron-dense layer of the cell wall facilitates the formation of the yeast cell wall from the inner layer of the hyphal cell wall [9, 34]. Direct budding of yeast-like elements from the hyphae wall always precedes yeast cell formation. Ultrastructural and biochemical studies on *P*. *brasiliensis* dimorphism demonstrated that modifications in cell wall glucans play a fundamental role in fungal differentiation and morphogenesis [28]. Enzymes that regulate the cell wall configuration during the *P*. *brasiliensis* mycelium-to-yeast transition, instead of being evenly distributed throughout the morphotypes, were distinctly prominent in the areas of fungal growth. Consistent with this observation, paracoccin was extremely concentrated in the growth regions of all *P*. *brasiliensis* morphotypes. In mycelia, paracoccin was concentrated in the fungal tips and lateral protrusions. This type of organization is considered essential for the delivery of growth factors and enzymes to the specific regions of the hyphae, contributing not only to the maintenance of cell wall integrity but also to chitin degradation and hyphal prolongation. In transition forms, paracoccin was detected in lateral swellings of differentiating hyphae from which chlamydoconidia arise. In yeasts, the presence of paracoccin in budding regions is also compatible with a role in cell growth. Corroborating with these results of PCN localization during the morphological transition process, we verified the PCN mRNA expression in all *P*. *brasiliensis* morphotypes and during the transition phases from hypha-to-yeast and yeast-to-hypha. It is worth mentioning the higher PCN transcript expression in hyphae, which is the fungal infecting phase, and during yeast-to-hypha transition. These results are coherent with the proposed direct involvement of PCN in the morphological transition process, and consequently, in the cell wall remodelling, as previously suggested [19].

In conclusion, this work provides support for the postulation that paracoccin plays a role in fungal growth and morphogenesis, participates in dimorphic fungal transformation, and, consequently, contributes to *P*. *brasiliensis* pathogenesis.

## Supporting information

S1 Fig Control of immunostaining reactions. We considered that to give a better idea of the PCN localization it was important to show the DIC images of the morphotypes from which the data was derived. *P*. *brasiliensis* hyphae (A–C) and yeasts (D–F) were incubated with chicken pre-immune IgY (A and D) and / or with the secondary antibody (B and E). The images show that there was no unspecific reaction. DIC microscopy of the stained samples (C and F). Similar results were obtained when used serum from pre-immune HIII mice (not shown).
(TIF)

S2 Fig Single staining for paracoccin and chitin localization during the initial transition of *P*. *brasiliensis* mycelium to yeast cells. Mycelia cultured in liquid medium were induced to undergo yeast transformation by shifting the temperature from 26°C to 37°C. Samples were harvested at 12 h (A–C), 24 h (D–F), and 48 h (G–I) after the temperature shift and immunostained for paracoccin (PCN), using chicken IgY anti-paracoccin antibody conjugated to Alexa Fluor 488 (green) and stained with WGA (red) for chitin, and with DAPI (blue) for DNA.
(TIF)

S3 Fig Single staining for paracoccin and chitin localization during the late transition of *P*. *brasiliensis* mycelium to yeast cells. Mycelia cultured in liquid medium were induced to undergo yeast transformation by shifting the temperature from 26°C to 37°C. Samples were harvested at 72 h (A–C), 96 h (D–E), and 120 h (F–G) after the temperature shift and immunostained for paracoccin (PCN), using chicken IgY anti-paracoccin antibody conjugated to Alexa Fluor 488 (green) and stained with WGA (red) for chitin, and with DAPI (blue) for DNA.
(TIF)

S4 Fig Single staining for localization of paracoccin and chitin in yeast cells of *P*. *brasiliensis*. *P*. *brasiliensis* yeast cells were stained for detection of paracoccin (A and C), using chicken IgY anti-paracoccin antibody conjugated to Alexa Fluor 488 (green), and for chitin (B and D) with Texas Red^®^-X WGA (red).
(TIF)
