# Supplementary figures and images for: Paracoccin distribution supports its role in Paracoccidioides brasiliensis growth and dimorphic transformation
Source: PLoS One. 2017 Aug 28;12(8):e0184010. doi: 10.1371/journal.pone.0184010 (PMC5573292; doi:10.1371/journal.pone.0184010)

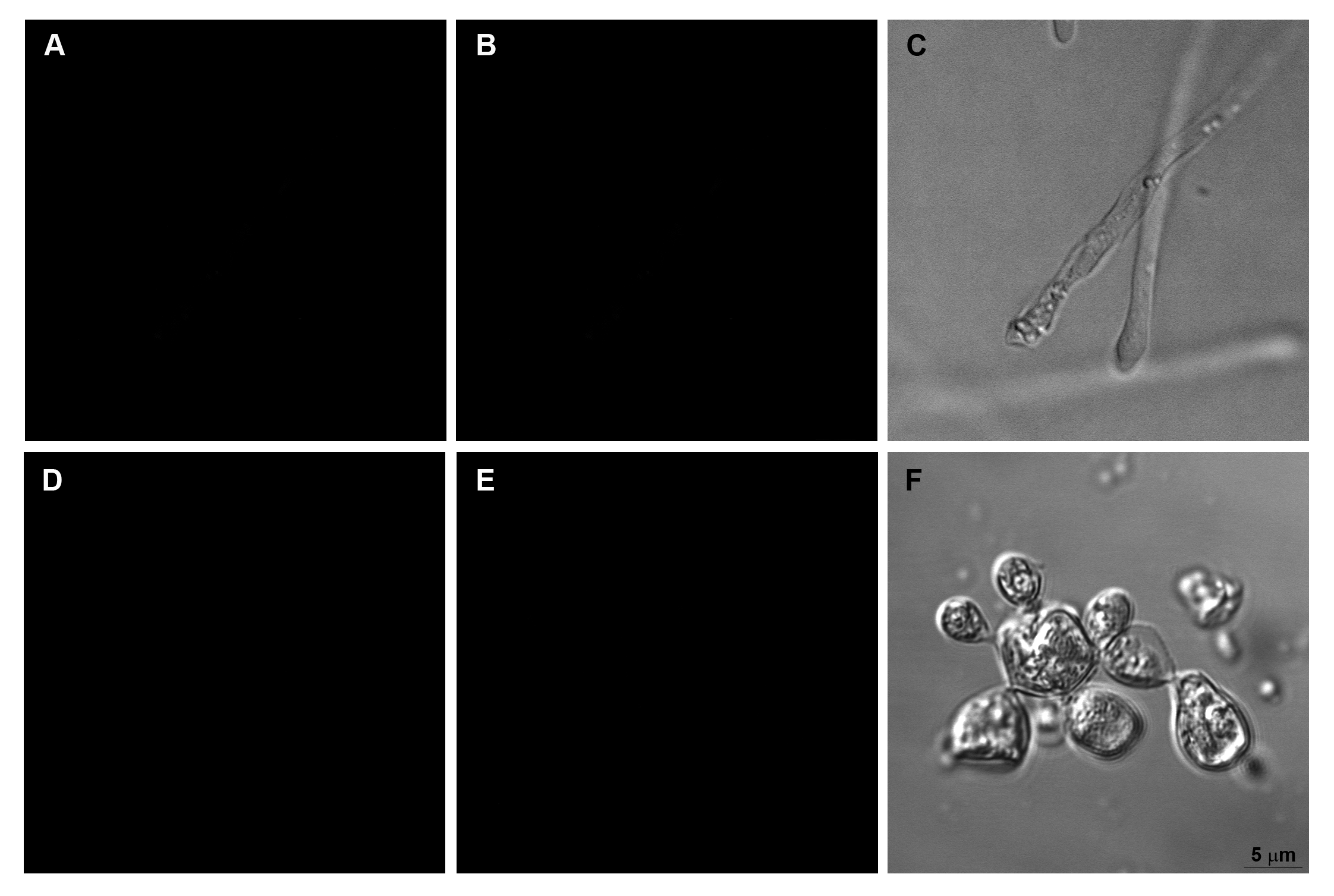

Supplement: S1 Fig — We considered that to give a better idea of the PCN localization it was important to show the DIC images of the morphotypes from which the data was derived. P. brasiliensis hyphae (A–C) and yeasts (D–F) were incubated with chicken pre-immune IgY (A and D) and / or with the secondary antibody (B and E). The images show that there was no unspecific reaction. DIC microscopy of the stained samples (C and F). Similar results were obtained when used serum from pre-immune HIII mice (not shown). (TIF) [file pone.0184010.s001.tif]

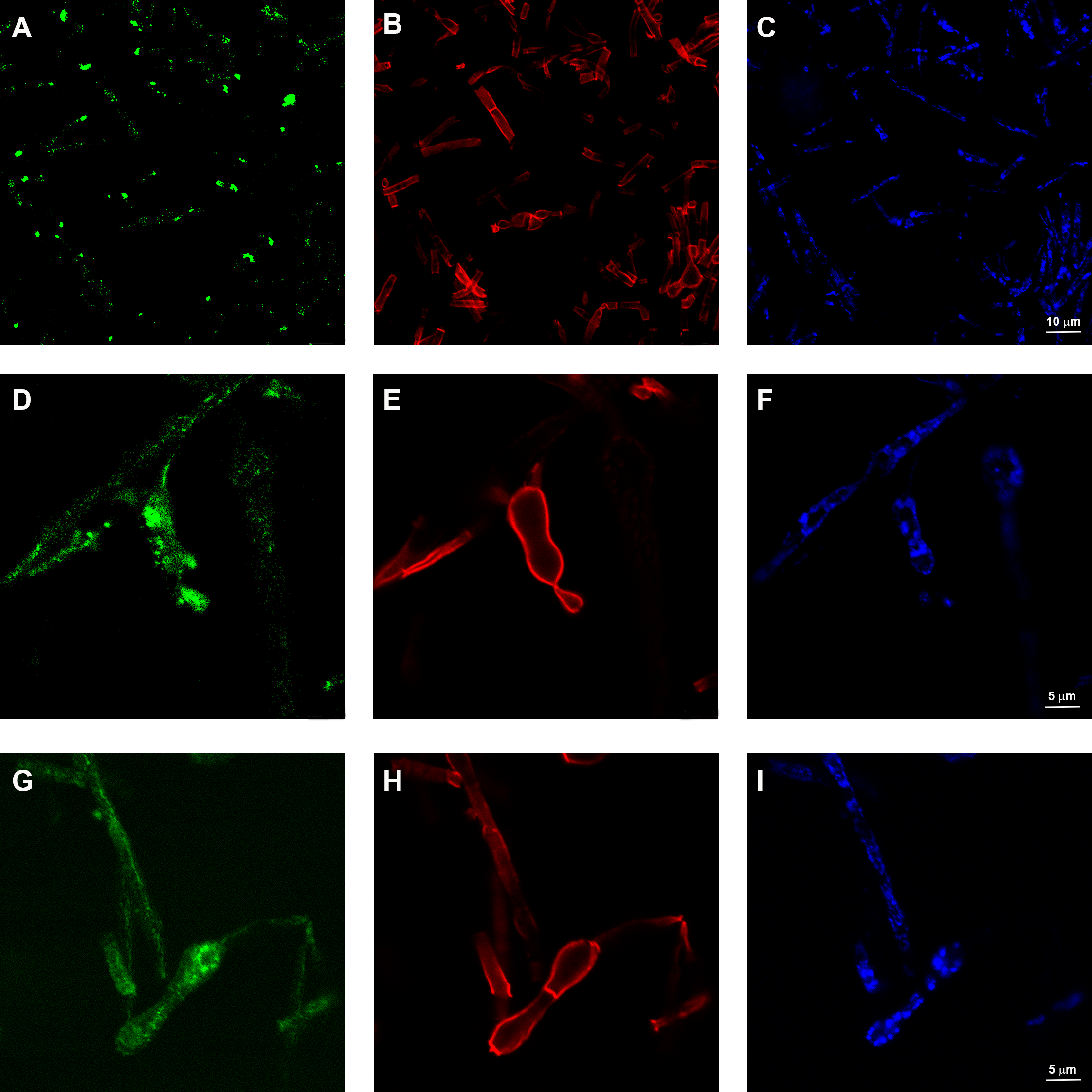

Supplement: S2 Fig — Mycelia cultured in liquid medium were induced to undergo yeast transformation by shifting the temperature from 26°C to 37°C. Samples were harvested at 12 h (A–C), 24 h (D–F), and 48 h (G–I) after the temperature shift and immunostained for paracoccin (PCN), using chicken IgY anti-paracoccin antibody conjugated to Alexa Fluor 488 (green) and stained with WGA (red) for chitin, and with DAPI (blue) for DNA. (TIF) [file pone.0184010.s002.tif]

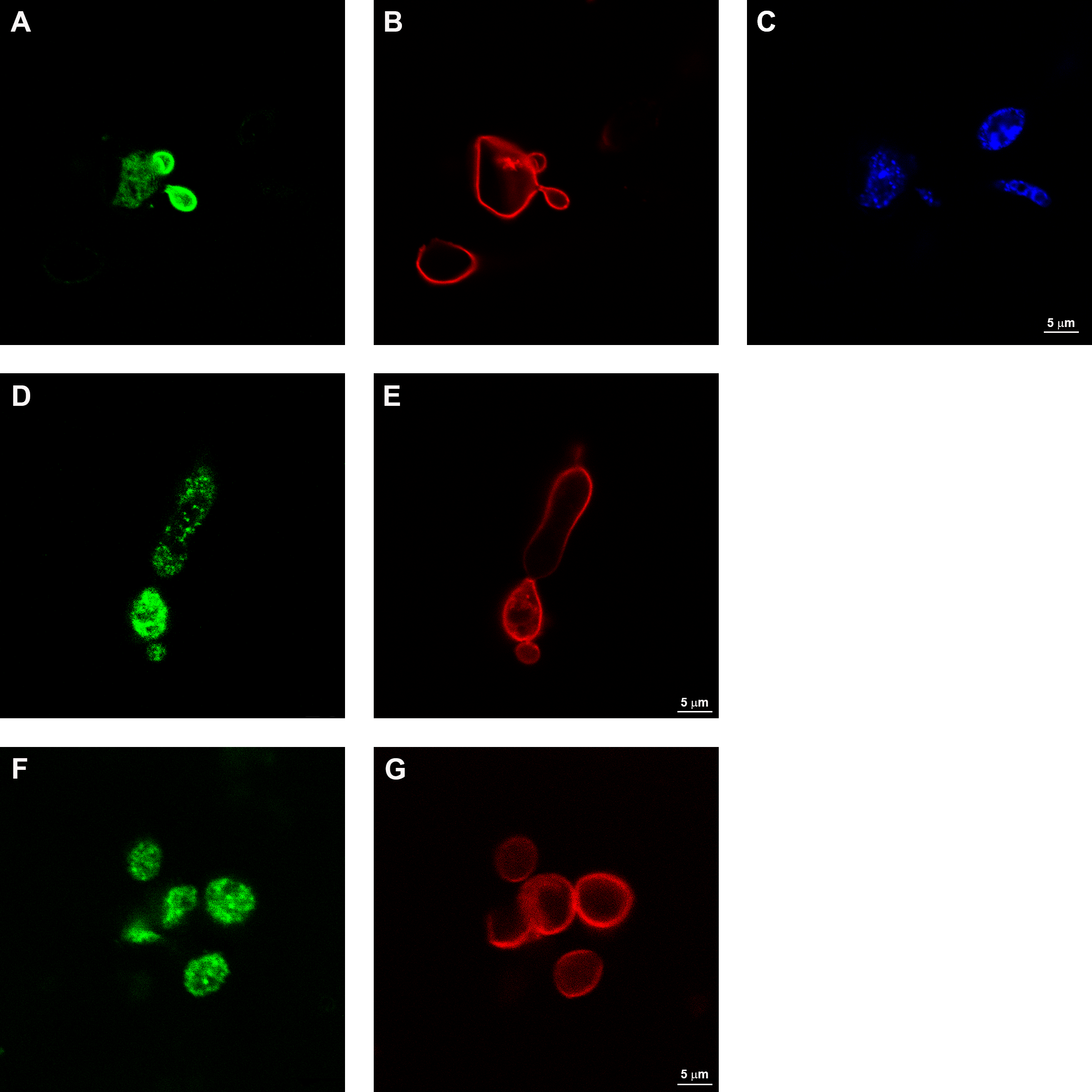

Supplement: S3 Fig — Mycelia cultured in liquid medium were induced to undergo yeast transformation by shifting the temperature from 26°C to 37°C. Samples were harvested at 72 h (A–C), 96 h (D–E), and 120 h (F–G) after the temperature shift and immunostained for paracoccin (PCN), using chicken IgY anti-paracoccin antibody conjugated to Alexa Fluor 488 (green) and stained with WGA (red) for chitin, and with DAPI (blue) for DNA. (TIF) [file pone.0184010.s003.tif]

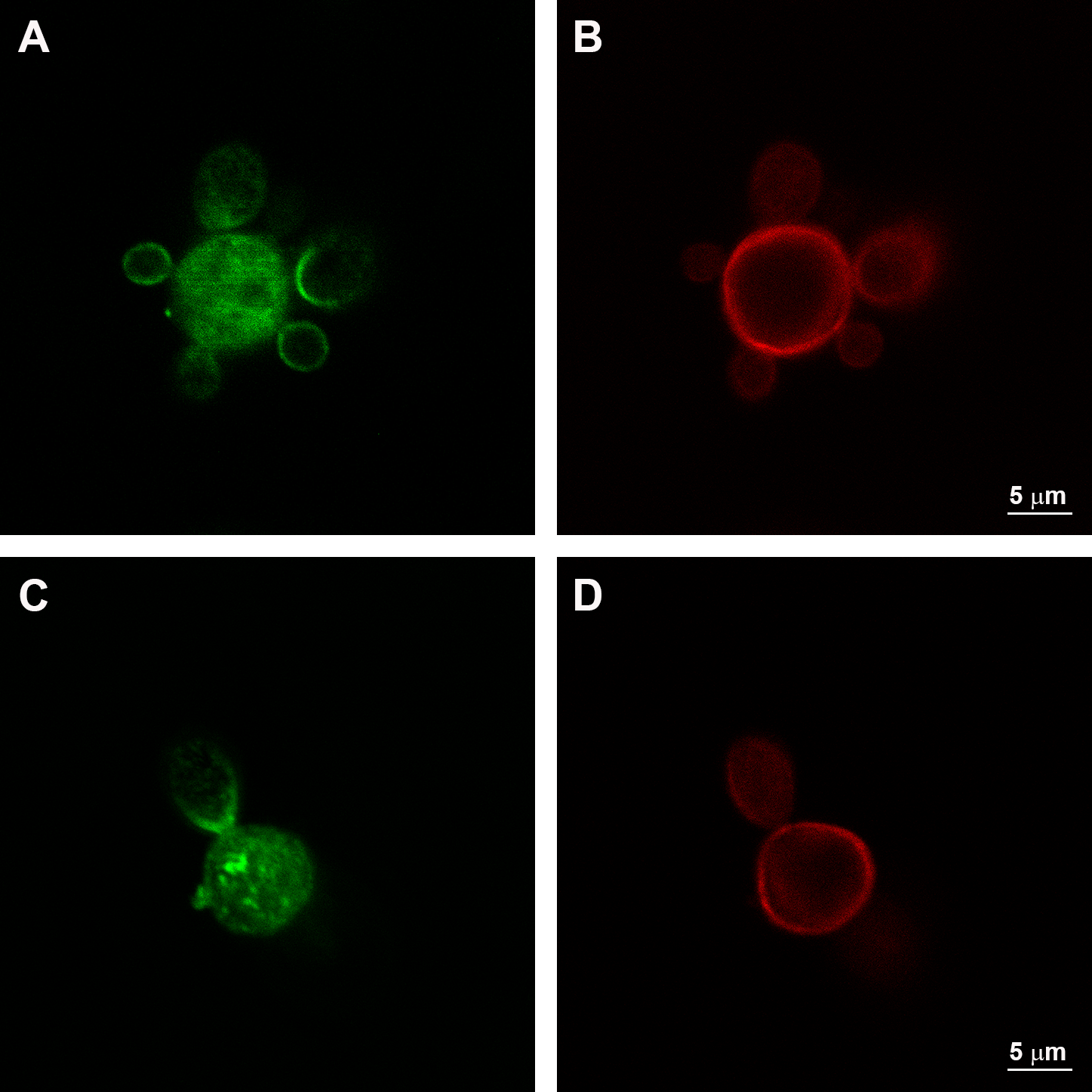

Supplement: S4 Fig — P. brasiliensis yeast cells were stained for detection of paracoccin (A and C), using chicken IgY anti-paracoccin antibody conjugated to Alexa Fluor 488 (green), and for chitin (B and D) with Texas Red®-X WGA (red). (TIF) [file pone.0184010.s004.tif]
